# Identification of Febuxostat as a New Strong ABCG2 Inhibitor: Potential Applications and Risks in Clinical Situations

**DOI:** 10.3389/fphar.2016.00518

**Published:** 2016-12-27

**Authors:** Hiroshi Miyata, Tappei Takada, Yu Toyoda, Hirotaka Matsuo, Kimiyoshi Ichida, Hiroshi Suzuki

**Affiliations:** ^1^Department of Pharmacy, The University of Tokyo Hospital, Faculty of Medicine, The University of TokyoTokyo, Japan; ^2^Department of Integrative Physiology and Bio-Nano Medicine, National Defense Medical CollegeTokorozawa, Japan; ^3^Department of Pathophysiology, Tokyo University of Pharmacy and Life SciencesTokyo, Japan

**Keywords:** allopurinol, BCRP, benzbromarone, bioavailability, drug-drug interactions, drug repositioning, topiroxostat, URAT1

## Abstract

ATP-binding cassette transporter G2 (ABCG2) is a plasma membrane protein that regulates the pharmacokinetics of a variety of drugs and serum uric acid (SUA) levels in humans. Despite the pharmacological and physiological importance of this transporter, there is no clinically available drug that modulates ABCG2 function. Therefore, to identify such drugs, we investigated the effect of drugs that affect SUA levels on ABCG2 function. This strategy was based on the hypothesis that the changes of SUA levels might caused by interaction with ABCG2 since it is a physiologically important urate transporter. The results of the *in vitro* screening showed that 10 of 25 drugs investigated strongly inhibited the urate transport activity of ABCG2. Moreover, febuxostat was revealed to be the most promising candidate of all the potential ABCG2 inhibitors based on its potent inhibition at clinical concentrations; the half-maximal inhibitory concentration of febuxostat was lower than its maximum plasma unbound concentrations reported. Indeed, our *in vivo* study demonstrated that orally administered febuxostat inhibited the intestinal Abcg2 and, thereby, increased the intestinal absorption of an ABCG2 substrate sulfasalazine in wild-type mice, but not in *Abcg2* knockout mice. These results suggest that febuxostat might inhibit human ABCG2 at a clinical dose. Furthermore, the results of this study lead to a proposed new application of febuxostat for enhancing the bioavailability of ABCG2 substrate drugs, named febuxostat-boosted therapy, and also imply the potential risk of adverse effects by drug-drug interactions that could occur between febuxostat and ABCG2 substrate drugs.

## Introduction

ATP-binding cassette transporter G2 (ABCG2) is a member of ABC transporter superfamily and is recognized as one of the most important drug efflux transporters (Giacomini et al., 2010; Lee et al., 2015; Mao and Unadkat, 2015). Following the identification of the expression of this protein as the cause of acquired multidrug resistance (MDR) in breast cancer cell lines, ABCG2 has been termed breast cancer resistance protein (Doyle et al., 1998). ABCG2 is expressed not only in cancer tissues but also in numerous normal tissues such as the small intestine and kidney (Doyle et al., 1998). Accumulating evidence indicates that ABCG2 plays a pivotal role as a gatekeeper against xenobiotics (Jonker et al., 2002; Giacomini et al., 2010).

ABCG2 regulates the pharmacokinetics and efficacy of its substrate drugs. In the small intestine, ABCG2 is involved in the efflux of its substrates from the epithelial cells into the intestinal lumen, which could lower their bioavailability. For instance, the plasma concentrations (Yamasaki et al., 2008) and efficacy (Wiese et al., 2014) of orally administered sulfasalazine, an antirheumatic drug, are modulated by the function of ABCG2, which appears to be affected by at least one SNP in its cognate gene *ABCG2* in humans. ABCG2 often lowers the bioavailability of other drugs such as rosuvastatin (Keskitalo et al., 2009; Tomlinson et al., 2010), which is widely used to treat dyslipidemia, and sunitinib (Mizuno et al., 2010), a multi-targeted receptor tyrosine kinase inhibitor used in cancer chemotherapy. The intestinal inhibition of ABCG2 would be an effective strategy to improve the efficacy of such drugs by enhancing their bioavailability. Therefore, the clinical inhibition of ABCG2 may be beneficial, although there are currently no appropriate drugs and candidates to inhibit ABCG2.

Recently, we and other research groups have independently found that ABCG2 is a physiologically important regulator of urate (Matsuo et al., 2009; Woodward et al., 2009; Ichida et al., 2012; Matsuo et al., 2014) as well as URAT1, a major component of the urate reabsorption system in the kidney and a target of hyperuricemia therapy (Enomoto et al., 2002). Hyperuricemia is defined as SUA levels > 7.0 mg/dL (Yamanaka, 2011) and is associated with some diseases such as metabolic syndrome, hypertension and gout (Richette et al., 2014). Genetically, decreased ABCG2 function is one of the major risk factors of hyperuricemia (Matsuo et al., 2009), since ABCG2 contributes to both intestinal and urinary excretion of urate from the human body into the feces and urine, respectively (Ichida et al., 2012; Matsuo et al., 2014). Thus, it is possible that increasing ABCG2 function could contribute to decreasing SUA levels in patients with hyperuricemia.

To date, safe modulation of ABCG2 function by chemicals in humans has not been achieved. Since both the inhibition and enhancement of ABCG2 function could have clinical consequences as described above, numerous efforts have been made to investigate and develop chemical compounds that interact with ABCG2. Historically, some promising ABCG2 inhibiting compounds, such as Ko143 (Allen et al., 2002) and elacridar (GF120918) (Hyafil et al., 1993), have been discovered, which were targeted at overcoming ABCG2-induced MDR. However, the efficacy and safety of these compounds in humans remain unclear, because, to our knowledge, their safety in humans has not been demonstrated in clinical studies. The similar problem is also the case for the new ABCG2 inhibitors developed recently (Juvale and Wiese, 2015; Ricci et al., 2016). Therefore, we aimed to identify a solution by exploring new promising agents for ABCG2 regulation from drugs currently available on the market. Since the approved drugs have a low risk of adverse effects in humans, this drug repositioning approach is expected to be highly feasible. In addition, based on the physiological function of ABCG2 as a urate transporter, we considered that some drugs that affect SUA levels (SUA-affecting drugs) might potentially interact with ABCG2. In this context, we chose the SUA-affecting drugs as a source of the screening library in this study.

The drugs investigated in this study were selected based on clinical reports demonstrating their SUA level altering effects in humans. The results of the *in vitro* investigation showed that 10 drugs potently inhibited ABCG2. Among them, febuxostat, a clinically used SUA-lowering drug, exhibited the strongest inhibitory effect on ABCG2 *in vitro*. We also revealed that febuxostat could enhance the intestinal absorption of sulfasalazine, a well-known ABCG2 substrate in both humans (Yamasaki et al., 2008) and mice (Zaher et al., 2006), by using WT and *Abcg2* KO mice. Our findings suggest novel potential applications and risks in clinical use of febuxostat.

## Materials and Methods

### Materials

The following compounds were purchased commercially from the sources indicated: allopurinol, benzbromarone, cyclosporine, D-fructose, elacridar, furosemide, hydrochlorothiazide, nicotinic acid, oxypurinol, rosuvastatin calcium salt, salicylic acid, 4-hydroxy chalcone (Wako Fine Chemical, Osaka, Japan); atorvastatin, chlorothiazide, febuxostat, mizoribine, pyrazinecarboxylic acid, ribavirin, tacrolimus, xylitol (Tokyo Chemical Industry, Tokyo, Japan); ethambutol, losartan (LKT Laboratories, St Paul, MN, USA); fenofibrate, probenecid, sulfasalazine, Ko143, ATP, AMP, creatine phosphate disodium salt tetrahydrate, creatine phosphokinase type I from rabbit muscle (Sigma-Aldrich, St. Louis, MO, USA); pyrazinamide (ACROS ORGANICS, Geel, Belgium); theophylline (Nacalai Tesque, Kyoto, Japan); and topiroxostat (MedChem Express, Princeton, NJ, USA). The [8-^14^C]-uric acid (53 mCi/mmol) was from American Radiolabeled Chemicals (St. Louis, MO, USA). All other chemicals used were commercially available and of analytical grade.

### Cell Culture

Human embryonic kidney 293 cell-derived 293A cells were purchased from Life Technologies (Carlsbad, CA, USA) and cultured in DMEM (Nacalai Tesque) supplemented with 10% fetal bovine serum (Biowest, Nuaillé, France), 1% penicillin/streptomycin, 2 mM L-glutamine (Nacalai Tesque), and 1 × Non-Essential Amino Acid (Life Technologies) at 37°C in an atmosphere of 5% CO_2_ as described previously (Toyoda et al., 2016a). All experiments were carried out with 293A cells at passages 10–20. To express human ABCG2 (NM_004827) fused with Myc-tag at its N-terminus (Myc-ABCG2) and EGFP (control), we used Myc-ABCG2 and EGFP-expressing adenoviruses constructed in our previous study (Ito et al., 2015), respectively. To express the URAT1, open reading frame of URAT1 (NM_144585.3) was cloned into a pcDNA3.1(+) vector (Life Technologies) with a FLAG tag at its N-terminus. To express mouse Abcg2 and EGFP (control), open reading frames of mouse Abcg2 (NM_011920) and EGFP were inserted into a pcDNA3.3 vector (Life Technologies), respectively.

### Animals

The FVB/NJcl WT mice were purchased from CLEA Japan (Tokyo, Japan). The *Abcg2* KO mice (Ichida et al., 2012) had been maintained in our laboratory as described previously. All the animals were housed in temperature- and humidity-controlled animal cages with a 12-h dark-light cycle, and free access to water and standard animal chow (MF, Oriental Yeast Company, Tokyo, Japan) as described previously (Ito et al., 2014; Takada et al., 2015). In the present study, all the *in vivo* experiments were conducted with 6–8-week-old male mice. The animal research protocols used in the present study were approved by the Animal Studies Committee of the University of Tokyo.

### *In vitro* Urate Transport Assay With ABCG2-Expressing Plasma Membrane Vesicles

The membrane vesicles were prepared from 293A cells infected with EGFP- or Myc-ABCG2- expressing adenovirus as described previously (Hayashi et al., 2005). Similarly, mouse Abcg2-expressing plasma membrane vesicles were prepared from 293A cells 48 h after the plasmid transfection using Polyethylenimine “MAX” (PEI-MAX) (1 mg/mL in milliQ water, pH 7.0; Polysciences, Warrington, PA, USA) as described previously (Stiburkova et al., 2016). The [8-^14^C]-urate transport assay with the ABCG2/Abcg2-expressing membrane vesicles was performed using a rapid filtration technique (Matsuo et al., 2009; Toyoda et al., 2016b). The urate transport activity was calculated as an incorporated clearance (μL/mg protein/min): (incorporated level of urate [DPM/mg protein/min]/urate level in the incubation mixture [DPM/μL]). ATP-dependent urate transport was calculated by subtracting the urate transport activity in the absence of ATP from that in the presence of ATP.

### Calculation of the Half-Maximal Inhibitory Concentration (IC_50_) Values and the Plasma Concentrations of Each Drug

The IC_50_ values of each test compound against the urate transport by ABCG2 were calculated as follows. First, the urate transport activities were measured in the presence of SUA-affecting drugs at several concentrations. The transport activities were expressed as a percentage of control (100%). Then, the calculated values were fitted to the following formula using the least-squares methods (Powles et al., 2007) with the Excel 2007 (Microsoft, Redmond, WA, USA) program:

$$\text{Predicted value}[\%]=100-\left(E_{\max}\times C^{n}/{IC}_{50}^{n}+C^{n}\right)$$

where, E_max_ is the maximum effect, C is the drug concentration, and n is the sigmoid-fit factor. The unbound concentrations of each drug in human plasma were calculated based on the general information provided by pharmaceutical industries. The unbound fraction in human plasma (f_u_) was calculated by subtracting the ratio of bound drugs from total drugs. The maximum unbound concentration of each drug in human plasma (f_u_C_max_) was calculated by multiplying f_u_ and the reported C_max_ together.

### *In vitro* Urate Uptake Assay in URAT1-Expressing 293A Cells

The 293A cells were seeded on a 12-well plate (Becton Dickenson & Co., Franklin Lakes, NJ, USA) at 2 × 10^5^ cells/well. Twenty-four hours after seeding, the cells were transiently transfected with the FLAG-URAT1 pcDNA3.1(+) vector or empty vector using PEI-MAX (0.4 μg plasmid/4 μL PEI-MAX/100 μL serum free DMEM/2 × 10^5^ cells) as described previously (Stiburkova et al., 2016). Forty-eight hours after the transfection, the cells were washed twice with Buffer T2 (125 mM Na-gluconate, 4.8 mM K-gluconate, 1.2 mM KH_2_PO_4_, 1.2 mM MgSO_4_, 1.3 mM Ca-gluconate, 25 mM HEPES, 5.6 mM D-glucose, and pH 7.4) and preincubated in Buffer T2 for 15 min at 37°C. Then, the buffer was exchanged to fresh Buffer T2 containing 2 μM [8-^14^C]-urate, and the cells were further incubated for the indicated periods. Subsequently, the cells were washed with ice-cold Buffer T2 twice and then lysed with 500 μL 0.2 M NaOH on ice with gently shaking for 1 h. The resulting lysates were transferred to 1.5-mL tubes, neutralized with 100 μL 1 M HCl, and then the radioactivity were measured using a liquid scintillator. The protein concentrations were determined using a bicinchoninic acid assay kit (Life Technologies) according to the manufacturer’s instruction. The urate transport activity was calculated as the incorporated clearance (μL/mg protein/min): (incorporated level of urate [DPM/mg protein/min]/urate level in the incubation mixture [DPM/μL]). URAT1-dependent urate transport activity was calculated by subtracting the urate transport activity of mock cells from that of the URAT1-expressing cells.

### Western Blot Analysis

The expression of Myc-ABCG2 on membrane vesicles and FLAG-URAT1 in transiently transfected-293A cells was confirmed using western blot analysis. To obtain the cell lysate samples of URAT1-expressing 293A cells, 48 h after the transfection, the cells were washed twice with phosphate-buffered saline without Ca^2+^ and Mg^2+^ and then dissolved in RIPA buffer (0.1% SDS, 0.5% deoxycholate, 1% NP40, 150 mM NaCl, 50 mM Tris-HCl, and pH 7.4) containing protease inhibitor cOmplete, EDTA-free (Roche, Basel, Switzerland). After centrifugation at 15,000 × *g* for 10 min at 4°C, the resulting supernatants were collected as the cell lysates. The membrane vesicles prepared from the adenovirus-infected 293A cells or cell lysates of vector-transfected 293A cells were subjected to 8.5% SDS-polyacrylamide gel electrophoresis and transferred to PVDF membranes (Immobilon, Millipore Corporation, Billerica, MA, USA). After blocking with 3% bovine serum albumin in 0.05% Tween 20 containing Tris-buffered saline for 1 h at room temperature, the membranes were treated with following antibodies as described previously (Matsuo et al., 2009); mouse anti-ABCG2 antibody (BXP-21) (Santa Cruz Biotechnology, Santa Cruz, CA, USA) (1:1000), a mouse anti-FLAG M2 antibody (Sigma Aldrich) (1:1000), a rabbit anti-Na^+^/K^+^-ATPase antibody (Santa Cruz Biotechnology) (1:1000), anti-mouse IgG or anti-rabbit IgG antibody labeled with horseradish peroxidase (GE Healthcare, Piscataway, NJ, USA) (1:2500). The immunoblotted membranes were treated with ECL Prime (GE Healthcare) and analyzed using a Chemidoc XRS (Bio-Rad Laboratories, Richmond, CA, USA).

### *In vivo* Abcg2 Inhibition Test

We conducted an *in vivo* Abcg2 inhibition test to examine the effect of febuxostat on the intestinal absorption of sulfasalazine, an ABCG2 substrate. Febuxostat was dissolved in 100 mM sodium bicarbonate buffer, pH 10. Prior to the experiment, the mice were fasted overnight, and then they were orally administered febuxostat by gavage at a dose of 150 mg/kg body weight (b.w.) with the expectation of complete inhibition of intestinal Abcg2. Twenty minutes later, sulfasalazine (20 mg/kg b.w.) was orally administered by gavage. At the indicated periods, the mice were anesthetized with diethyl ether and blood was collected from the jugular veins using heparinized syringes, followed by centrifugation at 3,000 × *g* for 10 min. The resulting supernatant (plasma) was collected and stored at -80°C until the LC/MS/MS analysis was performed.

### Measurement of Plasma Concentration of Sulfasalazine Using LC/MS/MS

The collected plasma was deproteinized with a fourfold volume of methanol containing 2 μg/mL 4-hydroxy chalcone as an internal standard. After vortexing for 10 min, the samples were centrifuged at 20,000 × *g* for 15 min at 4°C. Then, the supernatants were analyzed using the LC/MS/MS technique.

The LC/MS/MS analysis was conducted using an ultra-performance LC system connected to a Xevo TQ-S mass spectrometer (Waters Corporation, Milford, MA, USA). The samples were separated using a 1.7-μm particle ACQUITY C18 column (2.1 mm × 100 mm, Waters), maintained at 50°C, under gradient mobile phase conditions with a mixture of 0.1% formic acid in water and 0.1% formic acid in acetonitrile as solvents (0–1 min 70:30 v/v, 1–3 min 70:30 to 2:98 v/v, 3–5 min 2:98 v/v, and 5–6.5 min 70:30 v/v) with a flow rate of 0.3 mL/min. The separated samples were introduced into an MS in the positive and negative electrospray ionization mode for sulfasalazine and 4-hydroxy chalcone, respectively. Each compound was quantified in the multiple reactions monitoring mode (399.24 > 119.02, Cone 20 V, Collision 46 eV for sulfasalazine; 223.15 > 117.00, Cone 50 V, and Collision 36 eV for 4-hydroxy chalcone).

The AUC of sulfasalazine was calculated as the total area of the trapezoids formed by the points of the concentration and time in the concentration-time plots.

### Statistical Analysis

All statistical analyses were performed using the Excel 2007 program. Significant differences were identified using a two-way ANOVA followed by Tukey-Kramer *post hoc* test. The values were considered significant when *P* < 0.05.

## Results

### Effects of SUA-Affecting Drugs on the Urate Transport Activity of ABCG2 *In vitro*

**FIGURE 1:**
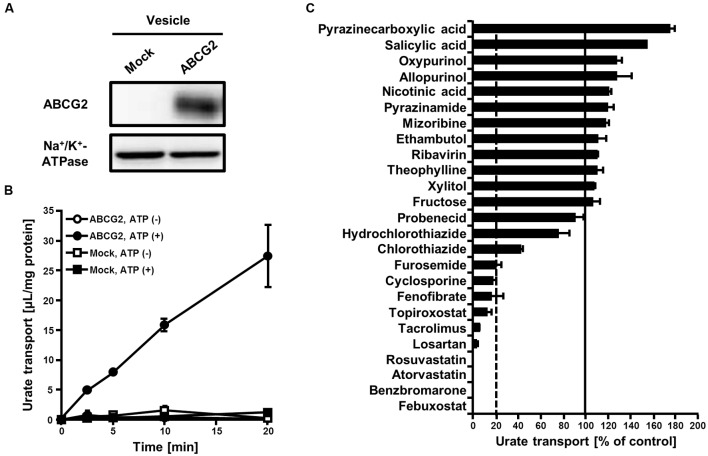
**Effect of serum uric acid (SUA)-affecting drugs on the urate transport activity of ABCG2.** **(A)** Expression of ABCG2 on membrane vesicles. Membrane vesicles (5 μg) were subjected to western blot analysis using a BXP-21, an anti-ABCG2 or anti-Na^+^/K^+^-ATPase antibody. **(B)** Time-dependent increase in the urate transport by ABCG2. The urate transport into membrane vesicles was measured at the indicated periods with (closed) or without (open) ATP. Values are expressed as mean ± SD. (*n* = 3). **(C)** The urate transport activities of ABCG2 in the presence of each SUA-affecting drug. Concentrations of each drug are shown in **Table 1**. The urate transport into membrane vesicles was measured in the presence of each SUA-affecting drug for 10 min. Data are shown as the percentage of vehicle control (without drugs). Values are expressed as mean ± SD (*n* = 3).

To examine the effects of 25 SUA-affecting drugs (**Table 1**) on ABCG2 function, we measured the urate transport activity of ABCG2 in the presence of each SUA-affecting drug. Expression of ABCG2 in the plasma membrane vesicles was confirmed using western blot analysis (**Figure 1A**). According to our previous reports (Matsuo et al., 2009; Stiburkova et al., 2016), we used 20 μM of urate in reaction mixtures in the following experiments. First, we examined the time-dependent increase in ATP-dependent urate transport into the ABCG2-expressing plasma membrane vesicle (**Figure 1B**). The urate transport activities of ABCG2 were higher than those of the mock (EGFP), and linearly increased for 20 min. Therefore, we examined the effect of SUA-affecting drugs on ABCG2 for 10 min at the indicated concentrations in **Table 1**. In this experiment, the concentrations of each drug (3–1000 μM) were determined as the maximum soluble levels in the transport buffer.

**Table 1:** List of SUA-affecting drugs in the present study.

Effect on SUA levels	Drugs	Maximum concentration in the present study [μM]	References
I Decrease expectedly	Allopurinol	300	Watts et al., 1965
	Benzbromarone	100	Enomoto et al., 2002
	Febuxostat	10	Osada et al., 1993
	Oxypurinol	100	Watts et al., 1965
	Probenecid	100	Gutman et al., 1954
	Topiroxostat	3	Okamoto et al., 2003
II Decrease unexpectedly	Atorvastatin^∗^	30	Ogata et al., 2010
	Fenofibrate	300	Harvengt et al., 1980
	Losartan	300	Kamper and Nielsen, 2001
	Rosuvastatin^∗^	100	Ogata et al., 2010
III Increase unexpectedly	Chlorothiazide	300	Kelley et al., 1973
	Cyclosporine^∗^	30	Palestine et al., 1984
	Ethambutol	1000	Postlethwaite et al., 1972
	Fructose	1000	Thomas et al., 1975
	Furosemide	300	Kelley et al., 1973
	Hydrochlorothiazide	1000	Healey et al., 1959
	Mizoribine	300	Ishikawa et al., 1986
	Nicotinic acid	300	Cruess-Callaghan and FitzGerald, 1966
	Pyrazinamide	300	Cullen et al., 1956
	Pyrazinecarboxylic acid	1000	Cullen et al., 1956
	Ribavirin	1000	Yamashita et al., 2008
	Salicylic acid	1000	Yu and Gutman, 1959
	Tacrolimus^∗^	30	Kanbay et al., 2005
	Theophylline	100	Yamamoto et al., 1991
	Xylitol	1000	Thomas et al., 1975


*^∗^Reportedly interact with ABCG2.*

The *in vitro* screening result revealed that 10 of the 25 drugs investigated decreased the urate transport activity of ABCG2 to <20% of that of the vehicle control (**Figure 1C**). Although 12 drugs tended to increase the urate transport activity of ABCG2, their effects were slight compared to the drastic inhibitory effects of the 10 drugs. Therefore, we focused on the most active 10 drugs in the further analyses.

### Febuxostat, an SUA-Lowering Drug Inhibited ABCG2 at Clinical Concentrations *In vitro*

**FIGURE 2:**
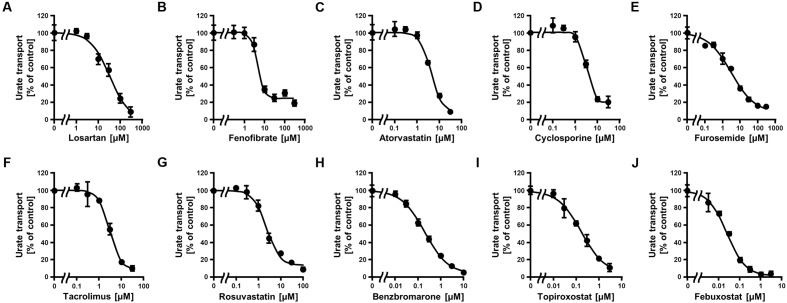
**Dose-dependent inhibition of ABCG2-mediated urate transport by serum uric acid (SUA)-affecting drugs.** The urate transport activities were measured in the presence of the following drugs at the indicated concentrations for 10 min. **(A)** Losartan, **(B)** Fenofibrate, **(C)** Atorvastatin, **(D)** Cyclosporine, **(E)** Furosemide, **(F)** Tacrolimus, **(G)** Rosuvastatin, **(H)** Benzbromarone, **(I)** Topiroxostat, and **(J)** Febuxostat. Data are shown as the percentage of vehicle control (without drugs). Values are expressed as mean ± SD (*n* = 3).

Since the *in vitro* screening was conducted in the presence of maximum concentrations of each drug, we subsequently estimated the clinical effects of the selected 10 drugs. First, to determine the IC_50_ values of each drug against the urate transport activity of ABCG2, we measured the urate transport activities in the presence of several concentrations of each drug (**Figure 2**). Then, the IC_50_ values were calculated from the results obtained using the least-squares methods (**Table 2**). Among the 10 drugs we examined, three (benzbromarone, topiroxostat and febuxostat) potently inhibited ABCG2 with IC_50_ values at submicromolar concentrations (0.20, 0.18, and 0.027 μM, respectively). Indeed, these values were considerably lower than those of four drugs that have been reported to interact with ABCG2, atorvastatin (4.3 μM), cyclosporine (3.2 μM), tacrolimus (3.1 μM) and rosuvastatin (2.3 μM).

**Table 2:** Calculated IC_50_ values for ABCG2-mediated urate transport activity and estimated f_u_, C_max_ and f_u_C_max_ values of SUA-affecting drugs.

Drugs	IC_50_ [μM]	f_u_	C_max_ [μM]	f_u_C_max_ [μM]	*In vivo* risk factor (f_u_C_max_/IC_50_)
Losartan	35	2.0 × 10^-2^	1.4	2.8 × 10^-2^	8.1 × 10^-4^
Fenofibrate	5.1	1.0 × 10^-2^	25	2.5 × 10^-1^	4.8 × 10^-2^
Atorvastatin	4.3	4.4 × 10^-2^	2.2 × 10^-3^	9.6 × 10^-5^	2.2 × 10^-5^
Cyclosporine	3.2	1.0 × 10^-1^	8.1 × 10^-1^	8.1 × 10^-2^	2.5 × 10^-2^
Furosemide	3.2	9.0 × 10^-2^	2.7	2.4 × 10^-1^	7.7 × 10^-2^
Tacrolimus	3.1	1.0 × 10^-2^	5.4 × 10^-2^	5.4 × 10^-4^	1.7 × 10^-4^
Rosuvastatin	2.3	1.2 × 10^-1^	9.4 × 10^-3^	1.1 × 10^-3^	4.8 × 10^-4^
Benzbromarone	0.20	3.7 × 10^-2^	5.4	2.0 × 10^-1^	9.9 × 10^-1^
Topiroxostat	0.18	2.5 × 10^-2^	7.0 × 10^-1^	1.7 × 10^-2^	9.9 × 10^-2^
Febuxostat	0.027	2.2 × 10^-2^	4.1	9.0 × 10^-2^	3.4


*The unbound fraction (f_*u*_), maximum concentration (C_*max*_) and maximum unbound concentration in human plasma (f_*u*_C_*max*_) values were calculated based on the information provided by pharmaceutical industries. As an *in vivo* risk factor, we determined the value of f_*u*_C_*max*_/IC_*50*_. The large value of this score reflects the possibility of ABCG2 inhibition by drugs in humans at clinical dose.*

To further characterize the potent inhibition of ABCG2 by febuxostat, we compared the activity of ABCG2 in the presence of febuxostat and two well-known ABCG2 inhibitors; Ko143, a fumitremorgin C analog, and elacridar (GF120918). In the presence of 100 nM of each compound, the urate transport activities of ABCG2 were 20 ± 3% with febuxostat, 75 ± 10% with Ko143 and 92 ± 10% with elacridar compared with the vehicle. These results indicate that the inhibitory effect of febuxostat against ABCG2 is stronger than those of these well-known ABCG2 inhibitors.

Next, we estimated the f_u_C_max_ based on the general information from pharmaceutical industries as described in Section “Materials and Methods” (**Table 2**), and calculated the ratio of f_u_C_max_/IC_50_, as an indicator of the possible *in vivo* clinical inhibition of ABCG2 by each drug. A high f_u_C_max_/IC_50_ value suggests that the drug has the potential to inhibit ABCG2 in humans. Among the 10 drugs we examined, benzbromarone and febuxostat had a relatively high f_u_C_max_/IC_50_ value (benzbromarone and febuxostat, 0.99 and 3.4, respectively). Since febuxostat had the highest f_u_C_max_/IC_50_ value and was expected to inhibit ABCG2 in humans, we proceeded to further analyze this drug.

### Febuxostat Drastically Increased Intestinal Absorption of Sulfasalazine in WT Mice, But Not in *Abcg2* KO Mice

**FIGURE 3:**
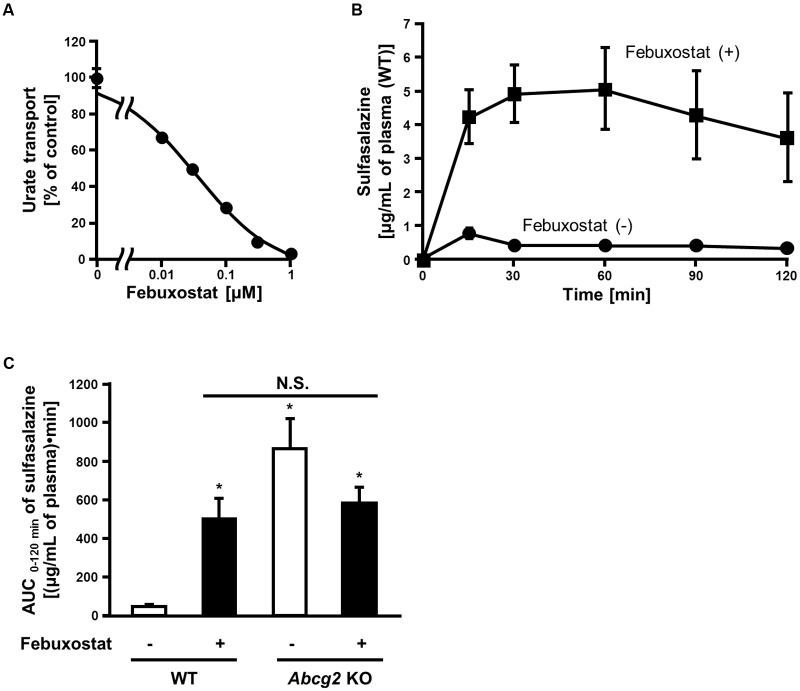
**Inhibition of mouse Abcg2 by febuxostat *in vitro* and *in vivo*.** **(A)** Dose-dependent inhibition of mouse Abcg2 by febuxostat *in vitro*. The urate transport activities of mouse Abcg2 were measured in the presence of febuxostat at the indicated concentrations. Data are shown as the percentage of vehicle control (without febuxostat). Values are expressed as mean ± SD (*n* = 3). **(B)** Time-dependent changes in sulfasalazine plasma concentration with the pre-administration of febuxostat. Twenty minutes after the oral administration of febuxostat (*n* = 4) or vehicle (*n* = 7), sulfasalazine was orally administered to WT mice. The blood samples were collected at the indicated periods, and the plasma concentrations of sulfasalazine were determined using LC/MS/MS. Values are expressed as mean ± SEM. **(C)** AUC _0-120 min_ of sulfasalazine in WT and *Abcg2* KO mice with or without pre-administration of febuxostat. Twenty minutes after the oral administration of febuxostat (*n* = 4 for both WT and *Abcg2* KO) or vehicle (*n* = 7 and 5 for WT and *Abcg2* KO, respectively), sulfasalazine was orally administered to WT and *Abcg2* KO mice. The blood samples were collected at 15, 30, 60, 90, and 120 min and then the plasma concentrations of sulfasalazine were determined using LC/MS/MS. AUC _0-120 min_ was determined for each group using the well-used trapezoidal rule. Values are expressed as mean ± SEM. Statistical analyses for significant differences were performed using two-way ANOVA followed by Tukey–Kramer method; ^∗^*p* < 0.05 *vs.* vehicle control WT mice; N.S., not significantly different among groups.

Our *in vitro* findings strongly suggested that febuxostat would inhibit ABCG2 *in vivo*. To examine this possibility, we investigated the inhibitory effect of febuxostat on the intestinal absorption of an ABCG2 substrate drug in WT and *Abcg2* KO mice. Prior to the *in vivo* analyses, we confirmed that febuxostat inhibited mouse Abcg2 *in vitro* in a similar manner to human ABCG2 as described above. The IC_50_ value of febuxostat against the urate transport activity of mouse Abcg2 was 35 nM (**Figure 3A**), which was comparable to that of human ABCG2 (IC_50_, 27 nM, **Table 2**), indicating that febuxostat inhibited mouse Abcg2 as strongly as it did human ABCG2.

To examine whether febuxostat inhibited Abcg2 *in vivo*, we focused on its effect on the absorption of sulfasalazine, an ABCG2 substrate, in the intestine where ABCG2 acts as a gatekeeper for xenobiotics. It is noteworthy that the intestinal absorption of sulfasalazine majorly depends on the ABCG2 function in both humans and mice (Zaher et al., 2006; Yamasaki et al., 2008). First, we addressed the time-dependent changes in the plasma concentration of orally administered sulfasalazine in WT mice. In WT mice pretreated with febuxostat, the plasma concentration of sulfasalazine was significantly higher than that in control mice at every time point (**Figure 3B**).

We then performed similar experiments using *Abcg2* KO mice and calculated the AUC of sulfasalazine. In WT mice, the administration of febuxostat increased the AUC of sulfasalazine (**Figure 3C**). On the other hand, in *Abcg2* KO mice, febuxostat did not increase the AUC of sulfasalazine. Moreover, there was no significant difference in the AUC of sulfasalazine among three groups; febuxostat-treated WT mice, vehicle-treated *Abcg2* KO mice and febuxostat-treated *Abcg2* KO mice. These results suggest that febuxostat enhanced the absorption of orally administered sulfasalazine by inhibiting Abcg2 *in vivo*.

### Febuxostat Hardly Affected URAT1-Mediated Transport of Urate

Some of the SUA-affecting drugs we investigated hardly affected ABCG2 function (**Figure 1C**), while their effects on other urate transporters remained unclear. Therefore, we focused on URAT1, another physiologically important urate transporter recognized as a pharmacological target of SUA-lowering drugs, and compared the effects of the drugs on ABCG2 and URAT1.

**FIGURE 4:**
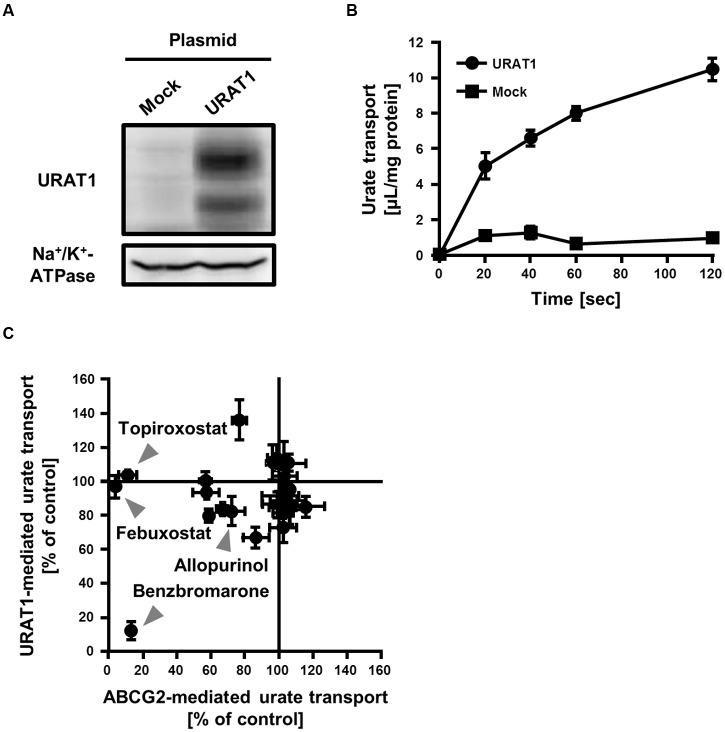
**Inhibitory effect of serum uric acid (SUA)-affecting drugs on the urate transport activities mediated by ABCG2 and URAT1.** **(A)** Expression of FLAG-URAT1 on 293A cells. Whole cell lysates (30 μg) of transient FLAG-URAT1-expressing 293A cells were subjected to western blot analysis using anti-FLAG or anti-Na^+^/K^+^-ATPase antibody. **(B)** Time-dependent increase of urate transport into FLAG-URAT1-expressing 293A cells. Values are expressed as mean ± SD (*n* = 3). **(C)** Effects of each SUA-affecting drug on the urate transport activities of ABCG2 and URAT1. The urate transport activities of ABCG2 and URAT1 were evaluated in the presence of 3 μM of each SUA-affecting drug. Data are shown as the percentage of vehicle control (without drugs). Values are expressed as mean ± SD (*n* = 3). Individual data for each drug are shown in **Supplementary Figure S1**.

To evaluate the effect of SUA-affecting drugs on the urate transport activity of URAT1, we established an *in vitro* assay system for the urate transport activity with 293A cells transiently expressing URAT1. The expression of URAT1 in the 293A cells was confirmed by western blot analysis (**Figure 4A**). Then, we measured the amount of [8-^14^C]-urate incorporated into URAT1-expressing 293A cells for 0–120 s (**Figure 4B**). The result showed that the urate transport activity in URAT1-expressing cells was more than fivefold higher than that in the mock cells, and the amount of incorporated urate increased in a time-dependent manner. Then, we evaluated the effect of drugs on the URAT1 activity for 20 s in subsequent investigations.

We compared the effect of each SUA-affecting drug (final concentration 3 μM) on the urate transport activity of ABCG2 and URAT1 (**Figure 4C**; **Supplementary Figure S1**). Among the 25 SUA-affecting drugs, only benzbromarone strongly inhibited both ABCG2 and URAT1. The urate transport activities of ABCG2 and URAT1 in the presence of 3 μM benzbromarone were 13 ± 2% and 12 ± 5%, respectively, compared to the vehicle control. On the other hand, febuxostat and topiroxostat did not affect the urate transport activity of URAT1, although they potently inhibited ABCG2. The rest of the investigated drugs had little effect on both ABCG2 and URAT1 at 3 μM.

## Discussion

In the present study, we examined the effects of 25 SUA-affecting drugs on the transport activity of ABCG2 in an attempt to identify a new chemical modulator of ABCG2 function from drugs on the market. The results of the *in vitro* screening identified 10 drugs that had inhibitory effects on ABCG2 function while the screening library did not contain drugs that strongly enhanced ABCG2 function. Further analyses demonstrated that febuxostat inhibited ABCG2 *in vitro* at clinical concentrations (**Figure 2**; **Table 2**) and *in vivo* in the murine intestine (**Figure 3**). Based on these *in vitro* and *in vivo* findings, it is possible that febuxostat has potential to inhibit ABCG2 in humans. It is worth noting that the inhibitory effect of febuxostat against ABCG2 was more potent than those of two well-known ABCG2 inhibitors, Ko143 and elacridar. This means that febuxostat has advantages not only in the safety but also in the inhibitory ability against ABCG2 as compared with those two ABCG2 inhibitors. Accordingly, in our opinion, febuxostat would be the most promising ABCG2 inhibitor in humans.

**FIGURE 5:**
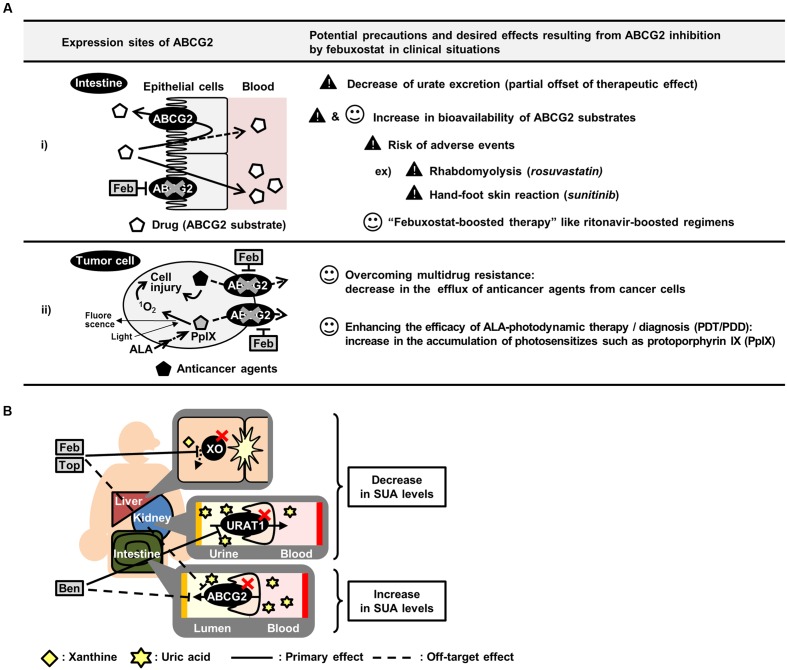
**Summary of potential effects of inhibiting ABCG2 *in vivo*.** **(A)** Schematic illustration of potential precautions and putative application of febuxostat as an ABCG2 inhibitor. **(B)** Schematic illustration of canceled out mechanism of benzbromarone, febuxostat and topiroxostat as SUA-lowering drugs. ALA, 5-aminolevulinic acid; Feb, febuxostat; Top, topiroxostat; Ben, benzbromarone.

The deliberate administration of febuxostat as an ABCG2 inhibitor could contribute to improving the pharmacokinetics and efficacy of ABCG2 substrate drugs (**Figure 5A**). Subjects with *ABCG2* SNPs, which lower ABCG2 function, reportedly exhibit higher bioavailability of ABCG2 substrates such as sulfasalazine (Yamasaki et al., 2008) and rosuvastatin (Keskitalo et al., 2009) than subjects with *ABCG2* WT. Therefore, febuxostat could induce a similar effect on the drug absorption in humans. In this regard, the present study provided supportive data demonstrating the febuxostat-dependent enhancement of intestinal absorption of sulfasalazine in mice (**Figure 3**). In pharmaceutical fields, taking advantages of inhibitory effect of ritonavir on CYP3A4, enhanced bioavailability of CYP3A4 substrate drugs is achieved by co-administration of ritonavir, which is known as ritonavir-boosting (Larson et al., 2014). Similarly, we propose here the possibility that febuxostat has considerable potential benefits in the form of a febuxostat-boosted therapy, which should be validated in future clinical investigations.

Moreover, the *in vivo* inhibition of ABCG2 could also be useful in cancer therapy (**Figure 5A**). Since *ABCG2* is one of the genes responsible for the development of MDR in cancer cells, the inhibition of ABCG2 could enhance the efficacy of chemotherapy, leading to an efficient accumulation of anticancer agents. In addition, ABCG2 is involved in the efflux of photosensitizers in 5-aminolevulinic acid-based photodynamic therapy (PDT) and photodynamic diagnosis (PDD) (Ishikawa et al., 2015; Palasuberniam et al., 2015). Considering that ABCG2 is expressed in cancer cells, febuxostat might enhance the efficacies of PDT/PDD by accumulating photosensitizers in the target cells.

Febuxostat-dependent impairment of ABCG2 function might have harmful effects as a risk factor for adverse events in combination medications (**Figure 5A**). For instance, while the efficacy of rosuvastatin would be enhanced by ABCG2 inhibition as described above, excess administration of rosuvastatin could induce rhabdomyolysis, a well-known severe adverse event (Magni et al., 2015). A similar concern has been raised with sunitinib, which could induce hand-foot skin reactions at high doses (Mizuno et al., 2010; Massey et al., 2015). Therefore, if febuxostat is co-administered with rosuvastatin or sunitinib and subsequently inhibits ABCG2, the bioavailability and plasma concentration of these drugs could be increased, resulting in an increased risk of adverse events. Hence, the physiological condition of the subjects administered febuxostat and ABCG2 substrate drugs should be monitored carefully. If the symptoms of adverse events are observed, a dose reduction of the ABCG2 substrate drugs should be considered.

Indeed, febuxostat, an SUA-lowering drug, could be clinically used with rosuvastatin and sunitinib. Rosuvastatin is a well-used drug in patients with dyslipidemia, likely to be complicated by hyperuricemia. Namely, there are numerous patients who are afflicted with these two lifestyle-related diseases, suggesting the possibility of simultaneous administration of rosuvastatin and febuxostat. In the case of sunitinib, we should mention the recent approval of febuxostat for the prevention of tumor lysis syndrome (TLS) in Europe and Japan. TLS is an oncogenic emergency resulting in metabolic disturbances including drastic increases in SUA levels. Considering the risk of TLS in chemotherapy with sunitinib (Nicholaou et al., 2007), the co-administration of febuxostat with sunitinib would increase in the near future.

The clinical prospects of febuxostat as an ABCG2 inhibitor should be confirmed by clinical investigations in future to compensate the limitation in the translational implication of the current study. Considering the diversity of substrate specificity in ABC transporters that is responsible for the multidrug efflux (Haimeur et al., 2004; Szakacs et al., 2006; Toyoda et al., 2008; Giacomini et al., 2010), the inhibitory effect of febuxostat on the other ABC transporters such as ABCB1 (known as P-glycoprotein) and ABCCs would be of interest. In the case of the development of ABCB1 inhibitors, despite the presence of a number of potential candidates, there have been no clinically approved substances because of their intolerable adverse effects and/or insufficient efficacy. Thus, clinical studies for the evaluation of the combination use of febuxostat should be carefully conducted with attention to unpredictable adverse events and its efficacy.

Focusing on urate kinetics, our results suggest that existing SUA-lowering drugs should have a room for improvement in efficacy. To develop more effective SUA-lowering drugs, the biological effect of the candidates should be carefully assessed not only on their primary molecular targets but also on other urate transporters or metabolic enzymes. Approved SUA-lowering drugs function as inhibitors of either xanthine oxidase (XO), a urate production enzyme, or URAT1. Allopurinol, the oldest XO inhibitor, has been used for patients with hyperuricemia over the last half-century (Watts et al., 1965), even though this drug has a risk of severe adverse events such as aplastic anemia (Arellano and Sacristan, 1993). The safety analyses of XO inhibitors in previous studies pointed out that both febuxostat and topiroxostat are superior to allopurinol (Matsumoto et al., 2011; Takano et al., 2005). However, the present study revealed the possibility that febuxostat and topiroxostat could inhibit ABCG2, resulting in the partial cancelation of their efficacy as a SUA-lowering drug (**Figure 5B**). Therefore, the SUA-lowering effects of these drugs might be weaker than expected because of their inhibition of ABCG2-mediated urate excretion. A similar inhibition pattern is also the case with benzbromarone, a URAT1 inhibitor (**Figure 5B**). Since allopurinol, febuxostat, topiroxostat and benzbromarone are the major SUA-lowering drugs presently known, the development of SUA-lowering drugs that do not inhibit ABCG2 would be the next strategy for developing a more effective hyperuricemia therapy.

## Conclusion

We demonstrated that febuxostat potently inhibited ABCG2 both *in vitro* and *in vivo*, suggesting that it could be the first clinical and safe ABCG2 inhibitor in humans. The possible drug–drug interaction between febuxostat and ABCG2 substrate drugs would have both beneficial and harmful effects. Our successful demonstration is an example of the promising beneficial effects, which provided the proof of concept for the potential of febuxostat-boosting. To ensure the safe clinical application of febuxostat, further investigations in humans would be important.

## Author Contributions

HsM, TT, and YT designed the research. HsM and YT conducted the all experiments. HsM, TT, and YT performed data analysis. All authors contributed to the writing and final approval of the manuscript.

## Conflict of Interest Statement

TT, HtM, KI, and HS have a patent pending. The other authors declare that the research was conducted in the absence of any commercial or financial relationships that could be construed as a potential conflict of interest.

## Abbreviations

ABC: ATP-binding cassette
ANOVA: analysis of variance
AUC: area under the blood concentration-time curve
C_max_: maximum drug concentration
CYP: cytochrome P450
DMEM: Dulbecco’s Modified Eagle’s Medium
DPM: disintegrations per minute
EGFP: enhanced green fluorescent protein
f_u_: unbound fraction
IC_50_: half-maximal inhibitory concentration
KO: knockout
LC/MS/MS: liquid chromatography-tandem mass spectrometry
MDR: multidrug resistance
PDD: photodynamic diagnosis
PDT: photodynamic therapy
SNP: single nucleotide polymorphism
SUA: serum uric acid
TLS: tumor lysis syndrome
URAT1: urate transporter 1
WT: wild-type
XO: xanthine oxidase
