# Supplementary figures and images for: Identification of Febuxostat as a New Strong ABCG2 Inhibitor: Potential Applications and Risks in Clinical Situations
Source: Front Pharmacol. 2016 Dec 27;7:518. doi: 10.3389/fphar.2016.00518 (PMC5187494; doi:10.3389/fphar.2016.00518)

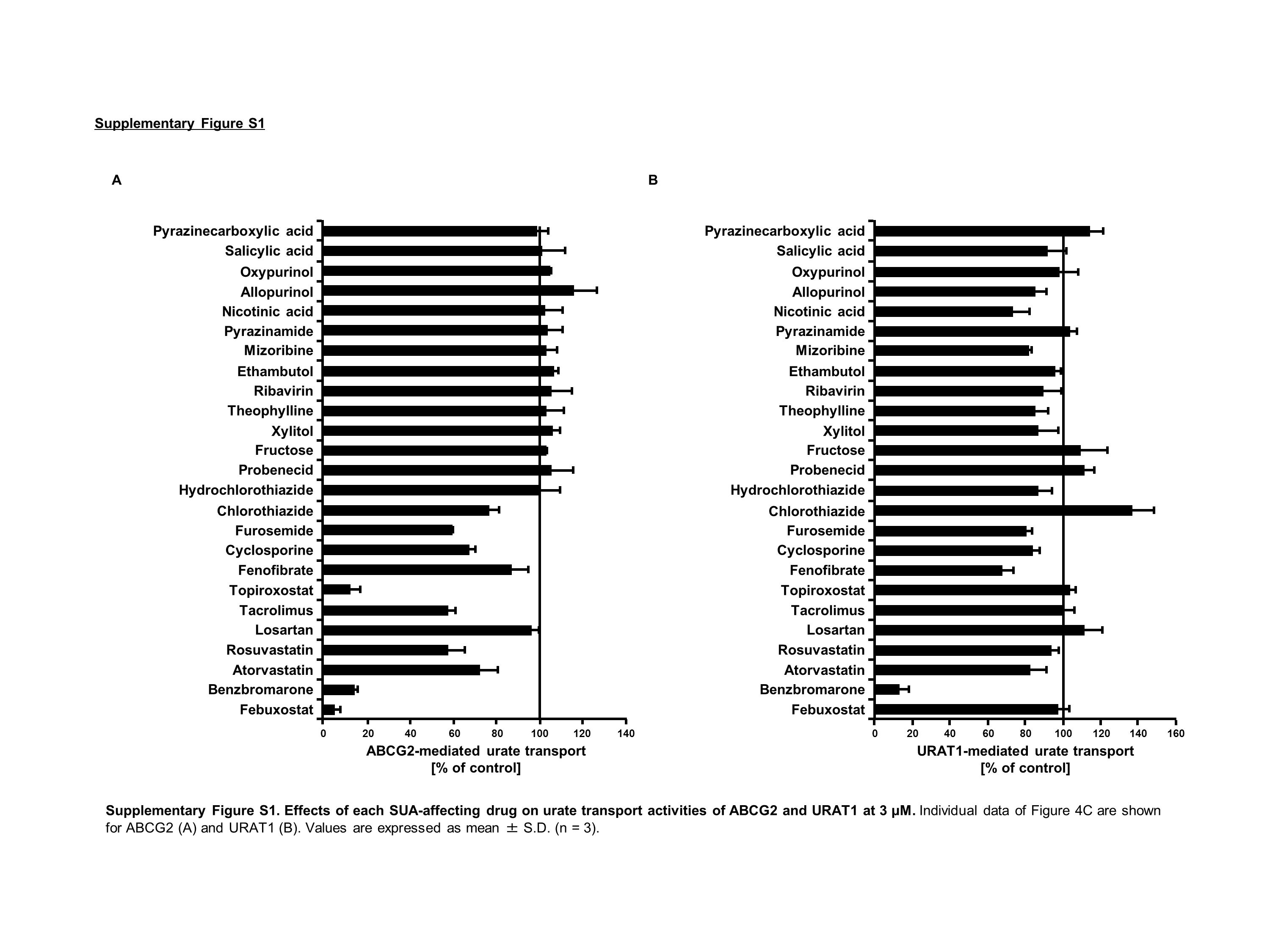

Supplement: Supplementary file 1 [file Image_1.TIF]
